# Host Nectin-1 Promotes Chlamydial Infection in the Female Mouse Genital Tract, but Is Not Required for Infection in a Novel Male Murine Rectal Infection Model

**DOI:** 10.1371/journal.pone.0160511

**Published:** 2016-08-03

**Authors:** Jessica A. Slade, Jennifer V. Hall, Jennifer Kintner, Regenia Phillips-Campbell, Robert V. Schoborg

**Affiliations:** Department of Biomedical Sciences, Center for Inflammation, Infectious Disease and Immunity, James H. Quillen College of Medicine, East Tennessee State University, Johnson City, Tennessee, United States of America; Midwestern University, UNITED STATES

## Abstract

*Chlamydia trachomatis* is the most common bacterial sexually transmitted pathogen, but more than 70% of patients fail to seek treatment due to the asymptomatic nature of these infections. Women suffer from numerous complications from chronic chlamydial infections, which include pelvic inflammatory disease and infertility. We previously demonstrated in culture that host cell nectin-1 knockdown significantly reduced chlamydial titers and inclusion size. Here, we sought to determine whether nectin-1 was required for chlamydial development *in vivo* by intravaginally infecting nectin-1^-/-^ mice with *Chlamydia muridarum* and monitoring chlamydial shedding by chlamydial titer assay. We observed a significant reduction in chlamydial shedding in female nectin-1^-/-^ mice compared to nectin-1^+/+^ control mice, an observation that was confirmed by PCR. Immunohistochemical staining in mouse cervical tissue confirmed that there are fewer chlamydial inclusions in *Chlamydia*-infected nectin-1^-/-^ mice. Notably, anorectal chlamydial infections are becoming a substantial health burden, though little is known regarding the pathogenesis of these infections. We therefore established a novel male murine model of rectal chlamydial infection, which we used to determine whether nectin-1 is required for anorectal chlamydial infection in male mice. In contrast to the data from vaginal infection, no difference in rectal chlamydial shedding was observed when male nectin-1^+/+^ and nectin-1^-/-^ mice were compared. Through the use of these two models, we have demonstrated that nectin-1 promotes chlamydial infection in the female genital tract but does not appear to contribute to rectal infection in male mice. These models could be used to further characterize tissue and sex related differences in chlamydial infection.

## Introduction

The obligate intracellular, bacterial pathogen *Chlamydia trachomatis* is responsible for over 100 million new infections each year and is the most prevalent curable STI in the world [1]. Despite the high prevalence of these infections, most genital tract infections with *C*. *trachomatis* serovars D-K are either symptomatically mild or asymptomatic [2]. Because asymptomatic patients do not seek treatment, chronic infections leading to long term complications are common. For example, as many as 40% of women with untreated *C*. *trachomatis* infections experience pelvic inflammatory disease (PID), which is also often subclinical [3]. *Chlamydia*-related PID is a major risk factor for tubal infertility and ectopic pregnancy [4]. The longer chlamydial infections go unnoticed, the more likely the chlamydiae are to ascend the genital tract and induce inflammation associated with these chronic illnesses.

Anorectal chlamydial infections are also a growing health concern for both men and women. Like urogenital chlamydial infections, these infections are largely asymptomatic; however, some irritation, pain, bleeding and/or discharge has been reported [5]. The prevalence of anorectal *Chlamydia* in men who have sex with men (MSM) and in women is similar, at approximately 9.8% and 9.5%, respectively [6]. However, as many as 85% of MSM experience rectal-only chlamydial infections, compared to 22% rectal-only infections in women [6]. Urogenital infections are slightly less prevalent than anorectal infections in MSM and occur in around 8% of those positive for chlamydial infections [7]. One study reported 8.8% detection of urethral infections and 20.2% rectal infections, but only 3.9% had co-occurring infections at both sites [8]. These data indicate that rectal-only infections represent a substantial number of chlamydial infections. Furthermore, as many as 34.4% of MSM with rectal chlamydial infections also have Human Immunodeficiency Virus (HIV) infection [7], suggesting that chlamydial infection could also increase the risk of HIV transmission in these patients. Though more studies are being conducted to establish the incidence and prevalence of rectal infections in various populations, little is known regarding *Chlamydia* pathogenesis within the human gastrointestinal tract. Because the GI tract may serve as a reservoir for chlamydial infection to exist long term and re-infect the female genital tract after anti-microbial therapy [9, 10], development of a murine model of chlamydial rectal infection seems warranted.

Chlamydiae engage in a unique biphasic developmental cycle. Upon entering the host, they exist in the infectious, non-replicative form called an elementary body (EB). The EB enter host cells and begin to differentiate into reticulate bodies (RB), the non-infectious, replicative form. This differentiation and replication occurs within a membrane bound vacuole called an inclusion. After a few rounds of division, the RB redifferentiate into EB and leave the host cell either by host cell lysis or extrusion of the chlamydial inclusion into the extracellular milieu to continue the infection [11, 12]. Chlamydiae can adapt to an unfavorable host environment by entering a developmental stage called persistence or the chlamydial stress response [13]. Under stressful *in vivo* and/or *in vitro* conditions the chlamydiae cease dividing but continue replicating their DNA. This causes the RB to become enlarged, or aberrant. Removal of the stressor allows the chlamydiae to re-enter and complete the normal developmental cycle [13, 14].

Nectin-1 belongs to a family of immunoglobulin-like molecules comprised of four members, nectin-1, -2, -3 and -4 [15]. Nectins are ubiquitously expressed and work in co-operation with other junctional proteins, called cadherins, to promote junction formation between cells. Nectins can be found in the adherens junctions (AJs) of polarized epithelial cells, at synapses in neurons and at points of contact between cultured epithelial cells [16]. Nectins are also important for maintaining contact between cells with specialized junctions, such as the pigment epithelial junctions in the ciliary body of the eye [17]. Several studies using nectin-1 transgenic mice have reported that in tissues where multiple nectins are expressed, no significant differences in AJs were noticed compared to wild type mice. These observations were attributed to the redundancy in function among nectins [17, 18]. Nectin-1 typically interacts with nectin-3 to form cell:cell junctions, but nectin-3 can also interact with nectin-2 [19]. Though compensation undoubtedly occurs, it may be incomplete. For example, in dental epithelium, nectin-1 and -3 are required for normal tooth development. In the absence of these two nectins, nectin-2 and nectin-4 interaction was sufficient for tooth development but overall tooth structure was abnormal [20].

Due to the ubiquitous expression of nectins, it is no surprise that nectin-1 also functions as a co-receptor for the Herpes Simplex Virus (HSV) gD protein on host epithelial cells and neurons [21]. HSV gD/nectin interaction also removes nectin-1 from the host cell surface [22, 23]. Previous work in our lab demonstrated that HSV super-infection of *Chlamydia* infected cells significantly reduced EB production [23, 24] and suggested that the loss of host nectin-1 was responsible for inhibition of chlamydial development [23]. We subsequently observed that that chlamydial inclusion size and production of infectious EB was significantly reduced in nectin-1 knockdown cells, confirming a role for host nectin-1 in chlamydial development–at least in culture [23].

Here we seek to determine the *in vivo* role of nectin-1 in chlamydial development. Based on our *in vitro* data, we hypothesized that nectin-1 is required for normal chlamydial development *in vivo*. We also present a novel male murine rectal infection model to study these severely understudied STIs. Interestingly, though nectin-1 was required to promote chlamydial infection in the female genital tract, nectin-1 is not required for chlamydial infection in a male mouse rectal infection model.

## Materials and Methods

### Ethics Statement

All animal experiments in this study were conducted in strict accordance with the National Institutes of Health “Guide for the Care and Use of Laboratory Animals”. The animal protocol (110602) was approved by the University Committee on Animal Care at East Tennessee State University under the guidelines of the Association for Assessment and Accreditation of Laboratory Animal Care, US Department of Agriculture, and in compliance with the Public Health Service Policy on Human Care and Use of Laboratory Animals.

### Cells and Bacteria

The cell line used was HeLa 229, a cervical adenocarcinoma epithelial cell line (ATCC No. CCL2.1). The *C*. *muridarum* Wiess strain was obtained from Kyle Ramsey (Midwestern University).

### Animal Handling and Genotyping

All mice were provided food and water ad libitum and kept on a standard 12-hour light/dark cycle. At the conclusion of each study, mice were euthanized via cervical dislocation.

**Fig 1:**
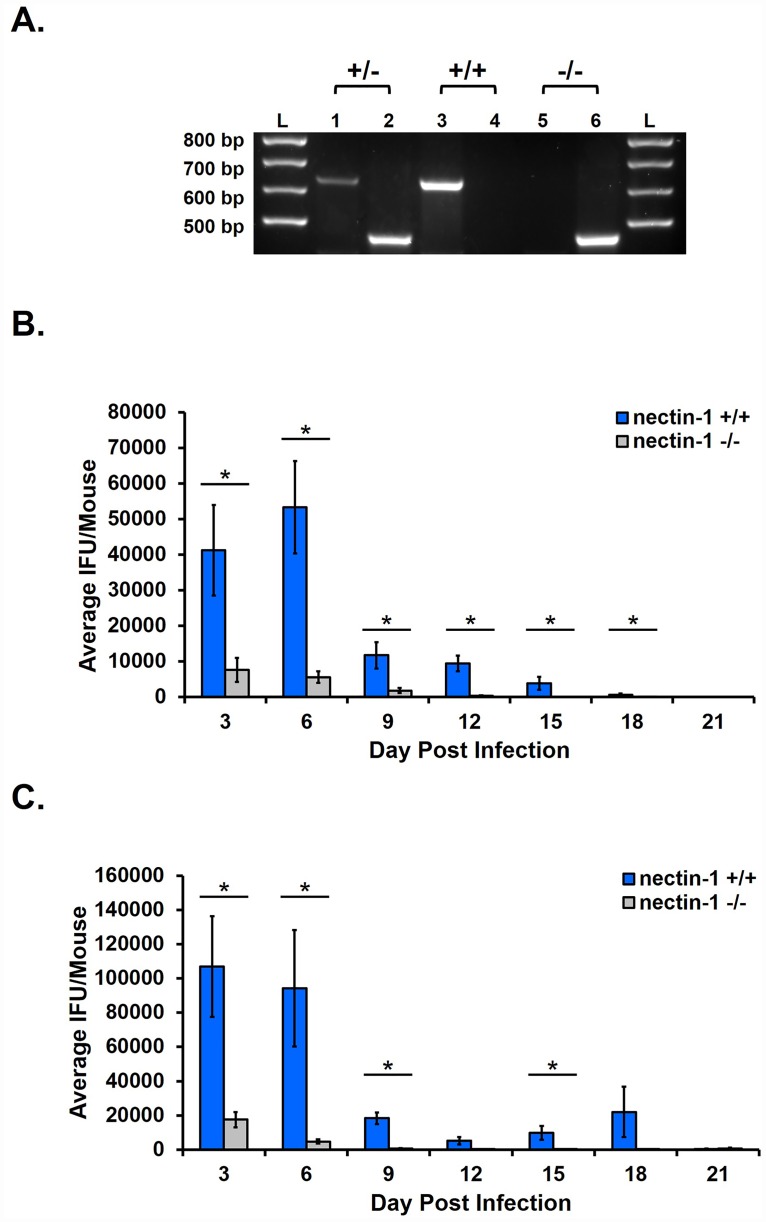
Host nectin-1 is required for chlamydial shedding in intravaginally infected mice. A) Example of genotypic characterization of nectin-1 heterozygous mice (lanes 1 and 2), nectin-1^+/+^ mice (lanes 3 and 4), and nectin-1^-/-^ mice (lanes 5 and 6). Molecular size ladders are represented by lanes labeled “L”. Nectin-1^+/+^ mice exhibit a single band at 639bp, nectin-1^-/-^ mice exhibit a single band at 459bp and heterozygotes exhibit bands at 639bp and 459bp. B and C) Mice were infected with either 1 x 10^3^ IFU (B) or 1 x 10^6^ IFU *C*. *muridarum* (C) on day 0. Swab samples from days 3 through 21 pi were used in chlamydial titer assays to determine chlamydial shedding. For panel B, n = 10 for the nectin-1^+/+^ group and n = 13 for the nectin-1^-/-^ group. For panel C, n = 19 per group. Shedding data are reported as the average IFU/mouse +/- SEM at each day pi. Shedding data are depicted as the combined data from 2 independent experiments each. Differences in shedding between groups at each day post shedding were determined with the unpaired Student’s t-test with p<0.05 considered significant, as indicated by an asterisk (*).

A nectin-1 knockout mouse colony was established from mice obtained from Jackson Laboratory (strain name B6.129X1(Cg)-Pvrl1^*tm1Ytk*^/J). Tail snips were obtained at 3–4 weeks of age and DNA was extracted using 75μl of NaOH. After a 1h incubation at 98°C, digested samples were neutralized with 75μl Tris HCL. Samples were vortexed briefly and centrifuged at 1200 x g for 3min. Supernatants were retained for use in genotyping. PCR was used to genotype mice from our nectin-1 colony using the MasterTaq kit (5 PRIME) and primers designed by Jackson Laboratories: nectin-1 forward CCG TAA AGG TCA AGG GCA GAG; nectin-1 wild type reverse GTG CCT GTC CCT TGT CCA; nectin-1 mutant reverse CTG TTG TGC CCA GTC ATA GCC. Each sample was subjected to 2 reactions, one using the nectin-1 forward and nectin-1 wild type reverse set, and a second reaction using the nectin-1 forward and the nectin-1 mutant reverse set. Nectin-1^+/+^ animals display one band at 639 base pairs (bp), nectin-1 ^-/-^ mice display one band at 459bp, and the heterozygotes display one of each band (Fig 1A). Male nectin-1^-/-^ and female nectin-1^+/-^ mice were used to maintain a breeding colony, producing a total of 1085 pups. Only 64 nectin-1^-/-^ females and 49 nectin-1^-/-^ males were produced, which were divided between the experiments described below and maintaining the breeding colony.

### Intravaginal Animal Infections

Female nectin-1^+/+^ and nectin-1^-/-^ mice were treated with 2.5mg Depo-Provera (Greenstone LLC, Peapack, NJ) by subcutaneous injection at 7–9 weeks of age. Mice were vaginally infected at 8–10 weeks of age with either 1 x 10^3^ or 1 x 10^6^ inclusion forming units (IFU) *Chlamydia muridarum* on day 0. The inocula were administered via micropipette in 10μl 2SPG. Mice were vaginally swabbed every 3 days through day 21 post infection (pi) as previously described [25]. Swabs were snap frozen in 2mL tubes containing 500μl 2SPG and three 3mm glass beads and stored at -80°C.

### Rectal Animal Infections

Male nectin-1^+/+^ and nectin-1^-/-^ mice aged 8–10 weeks were rectally infected with 20μl 2SPG containing 1 x 10^6^ IFU *C*. *muridarum*. The 20μl of inoculum was loaded into a tomcat catheter (Fisher) and a syringe loaded with 0.2cc air was connected. Mice were inoculated in the morning so that their intestines were empty of fecal matter. To inoculate, the catheter was inserted into the rectum to a depth of 3cm and the syringe was depressed gently. To monitor shedding, male mice were swabbed similarly to female mice. A calcium alginate swab was inserted rectally, rotated 15 times, collected into 500μL 2SPG, and snap frozen for use in chlamydial titer assays.

### Chlamydial Titer Assay

Swab samples were processed as previously described [25]. Briefly, samples were thawed, vortexed and sonicated. Dilutions were made for infection of HeLa 229 cells plated at 1 x 10^5^ cells per well on glass coverslips in duplicate wells of 24-well plates. Cultures were spin infected for 1h and refed with antibiotic/antifungal medium. For rectal swabs, the antibiotic/antifungal reagent concentrations were doubled. After a 24h incubation at 35°C, cells were fixed and permeabilized with methanol for 20min. Chlamydial inclusions were stained with Pathfinder anti-chlamydial LPS fluorescent stain (Bio-rad Laboratories, Hercules, CA) and total number of inclusions per coverslip was recorded. Infectious shedding was reported as average IFU/mouse +/- SEM. Chlamydial shedding between groups was analyzed by ANOVA and the Student’s unpaired t-test using Minitab 17. Values of p≤0.05 were considered significant (*).

### Immunohistochemistry

Genital tract tissue was removed and immediately fixed in 10% neutral buffered formalin. After 48h, tissues were placed in 70% ethanol until embedded in paraffin. Five μm cervical tissue sections were cut for use in ABC immunohistochemistry. Each slide contained 3–4 adjacent tissue sections. One slide from each mouse was used (5 mice per group from 2 independent infection experiments). For rectally infected male animals, colon tissue within 3cm depth from the rectum was removed, fixed, and paraffin embedded similarly to cervical tissue, except that colon tissue was embedded for longitudinal sectioning. A minimum of 30, 5 μm colon tissue sections were examined per mouse. Three wild type male murine colon tissues from a single experiment were harvested at day 24 and examined. Prior to staining, tissue sections were subjected to antigen retrieval using sodium citrate and were immersed in 0.3% H_2_O_2_ to quench endogenous peroxidase activity. Cervical and colon tissue sections were stained using a *C*. *trachomatis* primary antibody (1:1000, Abcam 31131) and the Vector Elite ABC kit (Vector Laboratories, PK-6101). For development, the Vector VIP peroxidase HRP substrate kit was used at a 1:2 dilution for 2min (Vector Laboratories SK-4600). The slides were counterstained for 2min with methyl green (Vector Laboratories H-3402). Positive staining is indicated by purple color. Cervical tissue was photographed at 40x magnification using a Zeiss Axiovert 40C microscope and color camera with Zen Blue imaging software. Colon tissue was photographed at 40x and 63x under oil immersion using a Zeiss Observer.Z1 and a black and white camera with Zen Blue imagining software.

### PCR and RT PCR

Total DNA and RNA were isolated from day 3 pi female swab samples as previously described using QIAmp DNA Blood Mini kit and RNA Easy Kit (Qiagen) [25]. To obtain cDNA, RNA was reverse transcribed using the QuantiTect Reverse Transcription kit (Qiagen) and RT- reactions were performed as appropriate. Primers previously reported by Phillips Campbell et al. were used to detect host β-actin, 16s DNA to quantify chlamydial genomes, and 16s rRNA to determine chlamydial viability [25]. Standard PCR conditions were applied except that PCR for β-actin and 16s rRNA were performed using an annealing temperature of 58°C. PCR for 16s DNA was performed using an annealing temperature of 65°C. All PCRs cycled 35 times. Amplification was performed with the MasterTaq Polymerase kit (5PRIME). After PCR, all reactions were electrophoresed as described previously [24]. Densitometry was used to quantify PCR amplification using the Genetools program (Syngene). All bands observed fell within the linear range of control template DNAs diluted from a range of 1:10 to 1:1000. For quantification of chlamydial genomes, amplification of 16s DNA was normalized to host β-actin. For quantification of 16s rRNA, the chlamydial genomes were normalized first to β-actin and then further normalized to 16s DNA for comparison of chlamydial viability between experimental groups as previously described [25]. Averages are reported as average band integrated intensity +/- SEM and were compared using the Student’s unpaired t-test. Values of p≤0.05 were considered significant (*).

## Results

### Nectin-1 is required for chlamydial shedding in the female murine genital tract

Through the use of nectin-1 knockdown HeLa cell lines, we previously demonstrated that the loss of nectin-1 resulted in significantly reduced chlamydial infectious EB production *in vitro*. To determine whether this phenomenon also occurred *in vivo*, we infected female nectin-1 wild type (nectin-1^+/+^) and nectin-1 knockout (nectin-1^-/-^) mice. Prior to infection, all mice were genotyped as described in the methods and demonstrated in Fig 1A.

Female nectin-1^+/+^ and nectin-1^-/-^ mice were intravaginally infected on day 0 and swabbed every three days until day 21 post infection (pi). Chlamydial titer assays were performed to enumerate average inclusion forming units (IFU) per group. To ensure that we were not using an inoculum so high as to overcome any effect the absence of nectin-1 may have on infection, we initially infected mice using a low inoculum of 1 x 10^3^ IFU/mouse. The nectin-1^-/-^ mice shed significantly fewer IFU compared to nectin-1^+/+^ mice on days 3, 6, 9, 12, 15 and 18 pi (Fig 1B). We then increased the inoculum to 1 x 10^6^ IFU *C*. *muridarum* to determine whether the reduction in chlamydial shedding could be overcome by increasing the amount of incoming chlamydiae. Interestingly, the nectin-1^-/-^ mice exhibited significantly reduced shedding on days 3, 6, 9, and 15 (Fig 1C) when infected with the higher inoculum. These data demonstrate that host nectin-1 is required for efficient chlamydial infection in the female mouse genital tract at both low (10^3^ IFU) and high (10^6^ IFU) inocula.

### Fewer chlamydial inclusions are detected in nectin-1^-/-^ mouse cervical tissue

**Fig 2:**
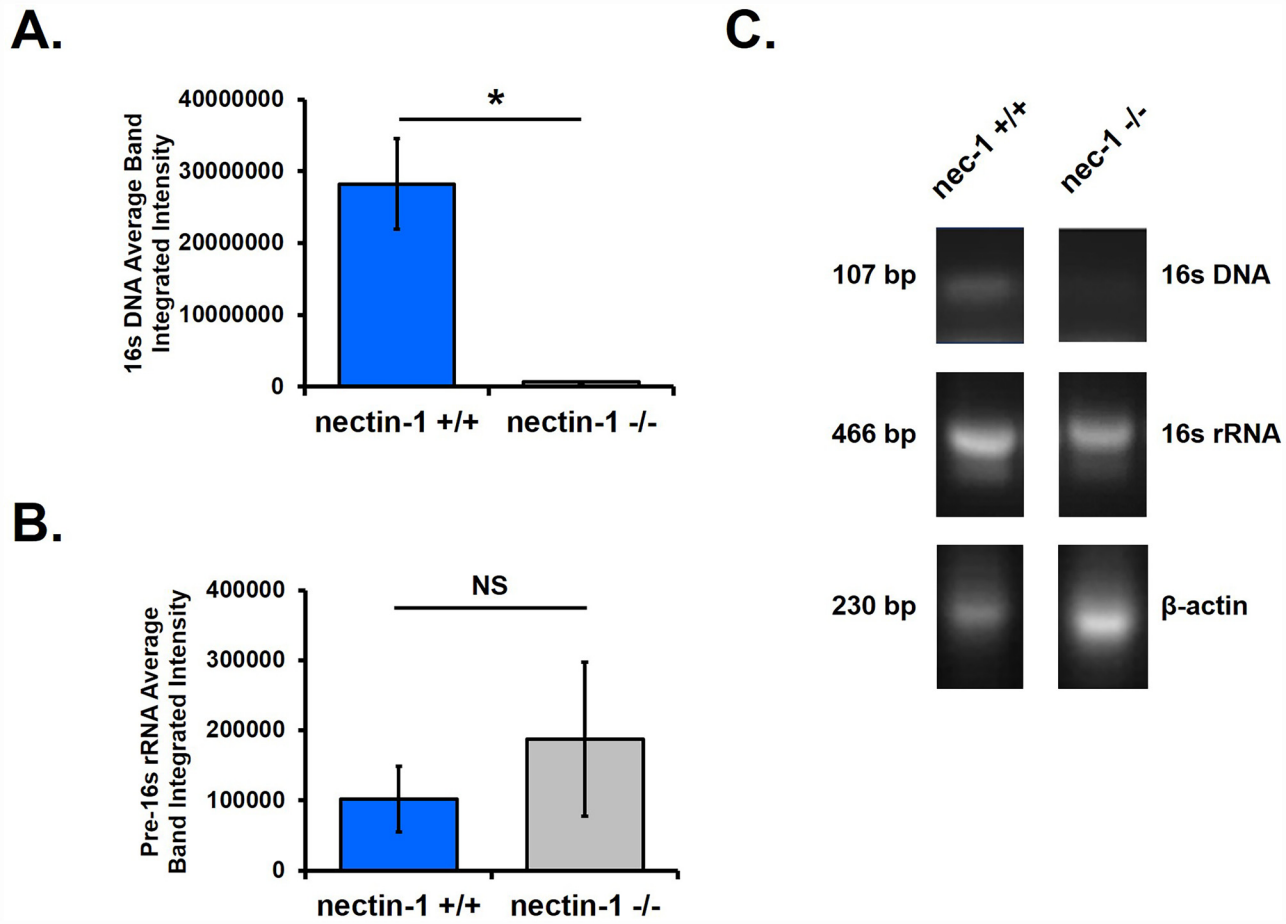
Nectin-1^-/-^ female mice have fewer detectable chlamydiae in the lower genital tract. A) Day 3 pi PCR semi-quantification of chlamydial genomes using 16s DNA normalized to host β-actin. Each group n = 4 and data are representative of 2 independent experiments. B) Day 3 pi PCR semi-quantification of chlamydial viability determined by amplification of chlamydial 16s rRNA normalized to chlamydial 16s DNA and host β-actin. A single data set was analyzed and n = 4 per group. Panels A and B are reported as average integrated intensity +/-SEM. Differences between groups were determined with the unpaired Student’s t-test with p<0.05 considered significant, as indicated by an asterisk (*). Non-significant comparisons are designated NS. C) Representative gel electrophoresis of chlamydial 16s DNA, chlamydial pre-16s RNA, and host β-actin PCR bands from one nectin-1^+/+^ and one nectin-1^-/-^ female mouse.

Because the reduction in chlamydial shedding in the nectin-1^-/-^ mice was so dramatic, we sought to further confirm our observations. First, PCR was performed using DNA and RNA extracted from swab samples at day 3 pi to quantify chlamydial genomes and chlamydial pre-16s rRNA as previously described [25]. Chlamydial genome quantity was significantly reduced in the nectin-1^-/-^ group compared to the nectin-1^+/+^ group (Fig 2A), confirming the previous vaginal shedding data. Additionally, no difference was observed in chlamydial pre-16s rRNA accumulation in the presence or absence of nectin-1 (Fig 2B). Representative chlamydial 16s DNA, chlamydial pre-16s RNA, and host β-actin PCR bands from one nectin-1^+/+^ and one nectin-1^-/-^ mouse are shown in Fig 2C. These data suggest that the small amount of detectable chlamydial DNA in nectin-1^-/-^ mice is from viable organisms and not from either dead organisms or leftover inoculum.

**Fig 3:**
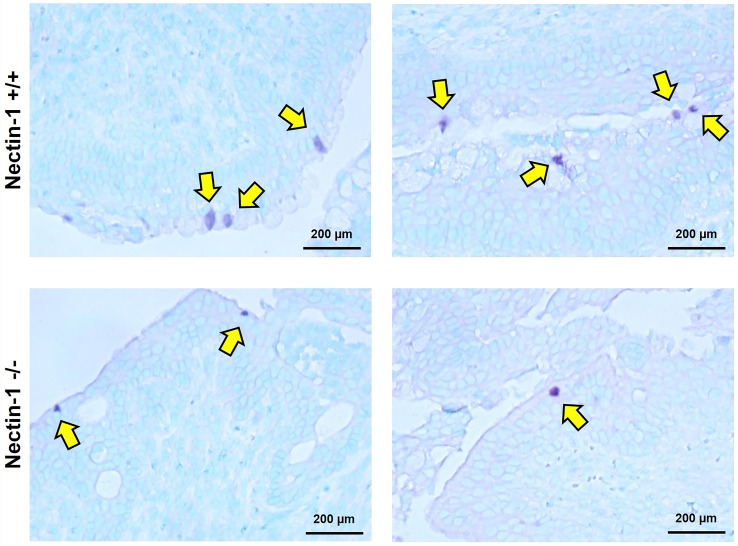
Nectin-1^-/-^ female mice have fewer detectable chlamydial inclusions in cervical tissue. A) Immunohistochemical staining of nectin-1^+/+^ (upper panels) and nectin-1^-/-^ (lower panels) *C*. *muridarum* infected cervical tissue harvested day 6 pi; n = 5 for each group. Yellow arrows indicate chlamydial inclusions. All mouse cervical samples were stained and cervical samples shown are from two individual mice per experimental group. Data are representative of two independent experiments.

We further sought to confirm that the chlamydiae were able to infect the cervical tissue. Female mice were infected with 1 x 10^6^ IFU *C*. *muridarum* on day 0 and swabbed on day 3 and 6 pi to confirm the reduction in chlamydial shedding described in Fig 1C. As previously observed, shedding was significantly lower in nectin-1^-/-^ animals compared to nectin-1^+/+^ animals as previously observed (840 IFU versus 2.1 x 10^4^ IFU at day 6pi; p<0.05). Mouse genital tracts from each group were removed after swabbing at day 6 pi and tissue sections immunohistochemically stained with a *C*. *trachomatis* antibody. In each group, inclusions were found only in the outermost epithelial layer and not in the deeper layers of the tissue. In the nectin-1^+/+^ animals, inclusions were not evenly dispersed along this epithelial layer, but appeared in small aggregates (Fig 3 upper panels). Locating inclusions in the nectin-1^-/-^ mice was challenging, as these aggregates were not observed. In many tissue sections from nection-1^-/-^ mice, no inclusions were observed. In sections from these animals that did stain for *Chlamydia*, typically only one inclusion was observed per mouse; however, one mouse had several observable inclusions (Fig 3 lower panels). Though we were unable to quantify the difference in inclusions observed in the cervical tissue between the nectin-1^-/-^ and nectin-1^+/+^ animals, far fewer inclusions were observed in the nectin-1^-/-^ group compared to the nectin-1^+/+^ group (Fig 3). These data indicate that *C*. *muridarum* can infect and form inclusions in the cervical epithelium of nectin-1^-/-^ mice, but to a lesser degree than observed in nectin-1^+/+^ animals. Overall, these data are consistent with the reduction in chlamydial shedding observed in Fig 1 and further suggest that there are fewer chlamydial inclusions in the cervical tissue of nectin-1^-/-^ mice.

### Nectin-1 is not required for chlamydial replication in a male murine rectal infection model

**Fig 4:**
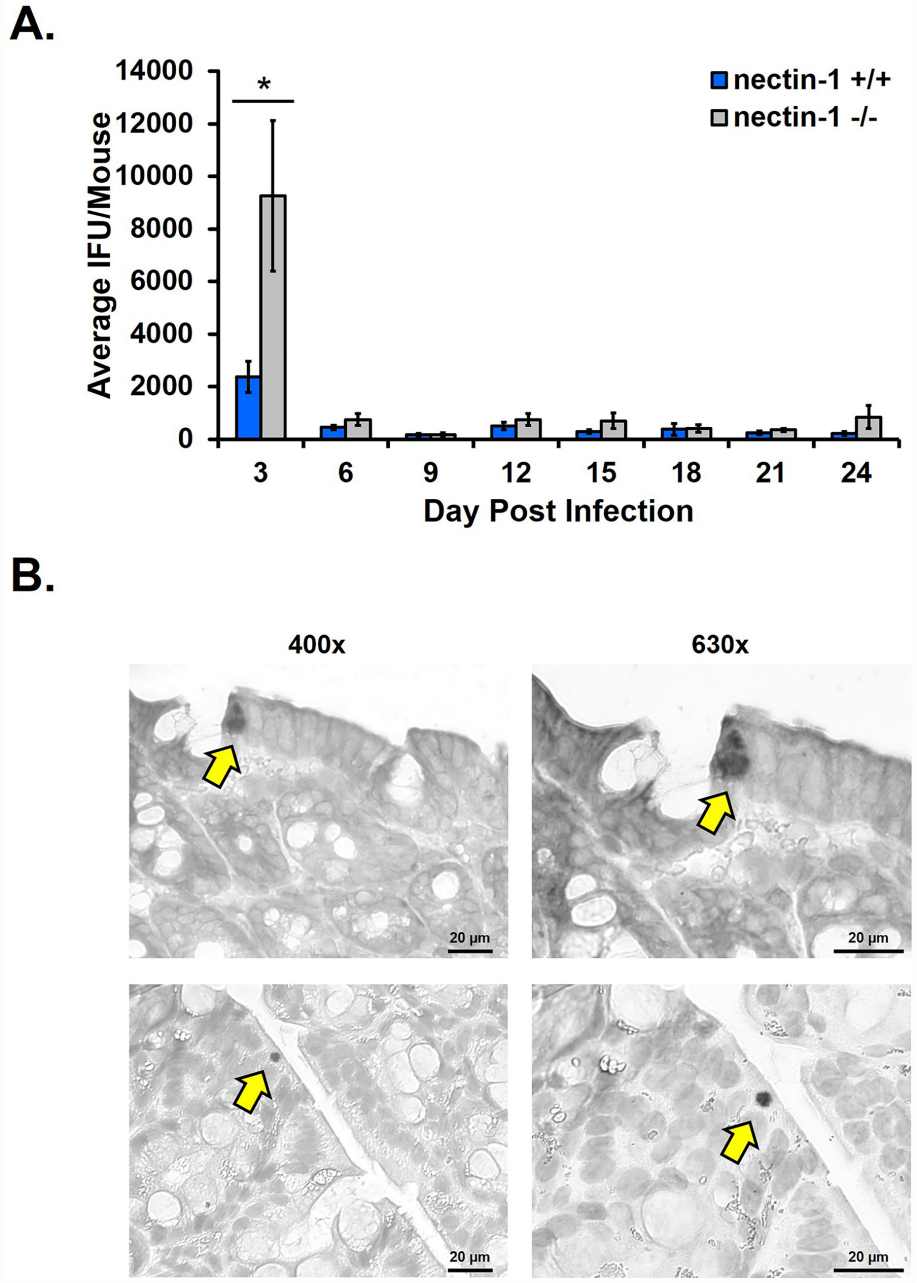
Nectin-1 is not required for male mouse rectal chlamydial infection. Male mice were rectally infected with 1x10^6^ IFU *C*. *muridarum* as described in the methods. A) Swab samples every 3 days from day 3 to 24 pi were used in chlamydial titer assays to determine chlamydial shedding. N = 16 and n = 18 for nectin-1^+/+^ and nectin-1^-/-^ groups, respectively. Shedding is reported as the average IFU/mouse +/- SEM at each day pi. Combined data from two independent experiments are shown. B) Immunohistochemical staining of male wild type colon tissue at 430x (left panels) and 630x (right panels). Yellow arrows indicate chlamydial inclusions. Colon tissue was harvested from *C*. *muridarum* rectally infected mice at day 24 pi; n = 3. Two representative inclusions are depicted from one wild type mouse. Data represent a single experiment.

The reduction in chlamydial infection observed in the female nectin-1^-/-^ mice led us to hypothesize that nectin-1 would also be required for other routes of chlamydial infection. Because there is evidence of spread from the female genital tract to the intestine [26] and because some male nectin-1^-/-^ mice were available for infection experiments, we sought to develop a male murine rectal infection model of chlamydial infection. Male nectin-1^+/+^ and nectin-1^-/-^ mice were rectally infected on day 0 then swabbed using the same technique established for vaginal chlamydial infections. Chlamydial titer assays were performed from swab samples obtained every 3 days until day 24 pi. In general, chlamydial detection was lower than that observed in the vaginal infection model, but shedding was maintained at a consistent level throughout the duration of the study, never reaching 0 IFU (Fig 4A). Using this model, significantly more chlamydial shedding was observed from nectin-1^-/-^ mice only on day 3 pi. Significant differences in rectal infection between male nectin-1^+/+^ and nectin-1^-/-^ mice were not observed at any other time point post-infection. Therefore, we conclude that nectin-1 is not required for rectal chlamydial infection in male mice since the absence of nectin-1 does not inhibit chlamydial infection at any time interval assayed.

Because shedding in male rectally infected mice was so much lower compared to female mice vaginally infected with the same inoculum (1 x 10^6^ IFU), we sought to determine whether *C*. *muridarum* was able to form inclusions in colon tissue. Yeruva *et al*. previously demonstrated that, in a murine *C*. *muridarum* oral infection model, rare inclusions were detectable in the intestine at day 25 pi in a single animal [10]. Similarly, when colon tissue was harvested at day 24 pi from three rectally infected male nectin-1 +/+ and -/- mice, chlamydial inclusions were detectable in only one animal, but multiple inclusions were found (Fig 4B). These data, which are similar to observations by Yeruva *at al*. [10], are also consistent with the low chlamydial titers obtained from rectally infected mice (Fig 4A). These data also demonstrate not only that chlamydial inclusions are able to form in rectally infected tissue, but that inclusions are detectable in the tissue as late as 24 days pi.

## Discussion

Here we demonstrated that the host cell adhesion protein nectin-1, is required for optimal chlamydial infection in the female mouse genital tract. Apart from forming junctions in co-operation with cadherins, nectins interact directly with the actin cytoskeleton through a protein called afadin, and indirectly through signaling events mediated by interactions with αvβ3 integrins [27]. Integrins associated with nectins stimulate activation of focal adhesion kinase (FAK) and Src, which ultimately leads to activation of Cdc42 and actin remodeling to form junctions [28]. Although Wyrick et al. used integrin-specific neutralizing antibodies to determine that integrins were not required for chlamydial entry, they did not determine whether chlamydial development or production of infectious EB were altered [29]. Additionally, cell culture experiments demonstrate that nectin-1 ablation has more effect on infectious EB development than on entry/inclusion formation [23]. Therefore, it remains possible that nectin/αvβ3 integrin interaction might be required for chlamydial development at some step after initial infection [29].

It is important to note that, αvβ3 integrins have been shown to interact exclusively with nectins-1 and -3 *in vitro*, but not nectins -2 and -4 [28]. This suggests that any compensation by nectin-2 to maintain cell junctions would not restore nectin-integrin signaling in nectin-1^-/-^ mice. Therefore, we speculate that in the absence of nectin-1, αvβ3 integrins are unable to associate with the appropriate nectins to induce cell signaling events required for actin cytoskeletal organization during inclusion maturation. Notably, chlamydiae recruit actin to the inclusion [11] and this appears to be required for inclusion expansion [30]. Association of actin with the inclusion is also required for extrusion-mediated EB exit from the host cell, but it appears that actin depolymerization from around the mature inclusion is required for chlamydial exit by lysis [11, 31, 32]. Regardless of the exit mechanism, these data suggest that nectin-integrin interactions might be critical to inclusion stability, development and possibly for subsequent release of infectious EBs.

Here we also present a novel male murine chlamydial rectal infection model. When considering the male wild type mice alone, our chlamydial titer results correspond with other models of gastrointestinal chlamydial infection. Upon oral infection in mice, peak detection of *C*. *muridarum* from cecal tissue lysates was observed at day 5 pi with 5 x 10^4^ IFU but held steady through day 75 pi with just under 10^4^ IFU detected on each sampling day [10]. Similarly, we also detected chlamydial shedding at a low but almost constant level throughout our 24 day time course, and shedding never reached 0 IFU (Fig 4A). Importantly, human rectal infections are a growing health concern. It has been estimated that treatment of anorectal *Chlamydia* with azithromycin is 83% efficacious compared to 97% efficacy in urogenital infections [33]. Patients may also contract an anorectal infection without being simultaneously infected by the oropharyngeal or urogenital route, a scenario likely far more common in MSM than women [6]. Our rectal model of chlamydial infection is thus a physiologically relevant mode of anorectal infection that could be used in future studies to further characterize the pathogenesis of these understudied chlamydial infections.

Contrary to our observations in the female nectin-1^-/-^ mice, male nectin-1^-/-^ mice rectally infected with 1 x 10^6^ IFU *C*. *muridarum* exhibited significantly increased chlamydial shedding compared to male nectin-1^+/+^ mice at day 3 pi; shedding thereafter was the same in each group (Fig 4A). Therefore, we conclude that nectin-1 is not required for rectal infection in male mice. These results were somewhat surprising, given the drastic reduction in infection observed in the female vaginal infection model. It seems likely that differences in sex and/or tissue type contribute to these divergent results. One possibility is that nectin-1 is expressed at very low levels, or not expressed, in rectal/gastrointestinal epithelial cells. Though nectins have been studied in colon and intestinal cancer cell lines, expression in normal human tissue has not yet been characterized [34–36]. In mice, nectin-2 and -3 are predominately expressed in the intestine, whereas nectin-1 and -4 are hardly detected [37]. This would be consistent with the low level of shedding observed during rectal/gastrointestinal tract infection, which is similar to the levels of vaginal shedding observed in nectin-1^-/-^ female mice (Fig 1 and [10]). In this case, deletion of the nectin-1 gene would make little difference in rectal shedding, since nectin-1 is low in those tissues already. If nectin-1 levels regulate chlamydial development, as our *in vitro* [23] and *in vivo* (Fig 1) observations suggest, this could explain the differences observed in GI and genital tract shedding.

It is also important to note that the female mice were hormone treated prior to infection, which prevents the mice from cycling and promotes chlamydial infection [38]. It has been demonstrated that both nectin-1 expression and chlamydial development can be altered by changes in estrogen and/or progesterone concentrations [39–42]. Since male mice are not hormone-treated before rectal infection, it is possible that nectin-1 expression in the GI tract would be enhanced, at least in wildtype mice, by hormone treatment. In humans, both males and females express estrogen and progesterone receptors in the intestine [43]. Furthermore, there is evidence to suggest that male GI tissue is responsive to progesterone [44]. Thus, in the future, we will determine whether nectin-1 is required for rectal chlamydial infections in female mice and whether hormone treatment affects infection outcome.

In this study, we determined that nectin-1 is required to promote intravaginal infection with *C*. *muridarum in vivo*. Because nectin-1 maintains interactions between adherens junctions and the actin cytoskeleton, it seems likely that the absence of nectin-1 prevents actin-dependent chlamydial development or affects the ability of *Chlamydia* to leave the host cell and ascend the genital tract. We also report, for the first time, establishment of an experimentally-tractable system in which to study the little understood pathogenesis, but growing incidence, of anorectal chlamydial infection. This model has allowed us to determine that host nectin-1 is not required for rectal infection in male nectin-1^-/-^ mice. Together, these two infection models may provide a better understanding of the differences in susceptibility, chlamydial infection outcome, and chronic disease that exists between men and women and in various tissue types.

## References

[1] World Health Organization (2011) Global incidence and prevalence of selected curable sexually transmitted infections 2008. http://apps.who.int/iris/bitstream/10665/75181/1/9789241503839_eng.pdf. Accessed 24 Feb 2015

[2] Marrazzo J, Suchland R (2014) Recent advances in understanding and managing Chlamydia trachomatis infections. F1000Prime Rep 6:120 10.12703/P6-120 25580274 PMC4251420

[3] Manavi K (2006) A review on infection with Chlamydia trachomatis. Best Pract Res Clin Obstet Gynaecol 20:941–51 16934531 10.1016/j.bpobgyn.2006.06.003

[4] Paavonen J (2004) Sexually transmitted chlamydial infections and subfertility. Int Congr Ser 1266:277–286

[5] van Liere GAFS, Hoebe CJPA, Niekamp A-M, Koedijk FDH, Dukers-Muijrers NHTM (2013) Standard symptom- and sexual history-based testing misses anorectal Chlamydia trachomatis and neisseria gonorrhoeae infections in swingers and men who have sex with men. Sex Transm Dis 40:285–9 10.1097/OLQ.0b013e31828098f8 23486492

[6] van Liere GAFS, van Rooijen MS, Hoebe CJPA, Heijman T, de Vries HJC, Dukers-Muijrers NHTM (2015) Prevalence of and Factors Associated with Rectal-Only Chlamydia and Gonorrhoea in Women and in Men Who Have Sex with Men. PLoS One 10:e0140297 10.1371/journal.pone.0140297 26513479 PMC4626043

[7] Patton ME, Kidd S, Llata E, Stenger M, Braxton J, Asbel L, et al (2014) Extragenital gonorrhea and chlamydia testing and infection among men who have sex with men—STD Surveillance Network, United States, 2010–2012. Clin Infect Dis 58:1564–70 10.1093/cid/ciu184 24647015 PMC4666527

[8] Huffam S, Chow EPF, Fairley CK, Hocking J, Peel J, Chen M (2015) Chlamydia infection in individuals reporting contact with sexual partners with chlamydia: a cross-sectional study of sexual health clinic attendees. Sex Transm Infect 91:434–9 10.1136/sextrans-2015-052068 26056390

[9] Annan NT, Sullivan AK, Nori A, Naydenova P, Alexander S, McKenna A, et al (2009) Rectal chlamydia—a reservoir of undiagnosed infection in men who have sex with men. Sex Transm Infect 85:176–9 10.1136/sti.2008.031773 19176570

[10] Yeruva L, Spencer N, Bowlin AK, Wang Y, Rank RG (2013) Chlamydial infection of the gastrointestinal tract: a reservoir for persistent infection. Pathog Dis 68:88–95 10.1111/2049-632X.12052 23843274 PMC3751173

[11] Chin E, Kirker K, Zuck M, James G, Hybiske K (2012) Actin recruitment to the Chlamydia inclusion is spatiotemporally regulated by a mechanism that requires host and bacterial factors. PLoS One 7:e46949 10.1371/journal.pone.0046949 23071671 PMC3469565

[12] Wyrick PB (2000) Intracellular survival by Chlamydia. Microreview. Cell Microbiol 2:275–282 11207584 10.1046/j.1462-5822.2000.00059.x

[13] Hogan RJ, Mathews SA, Mukhopadhyay S, Summersgill JT, Timms P (2004) Chlamydial persistence: beyond the biphasic paradigm. Infect Immun 72:1843–55 15039303 10.1128/IAI.72.4.1843-1855.2004 PMC375192

[14] Schoborg R V. (2011) Chlamydia persistence—a tool to dissect chlamydia-host interactions. Microbes Infect 13:649–662 10.1016/j.micinf.2011.03.004 21458583 PMC3636554

[15] Sakisaka T, Takai Y (2004) Biology and pathology of nectins and nectin-like molecules. Curr Opin Cell Biol 16:513–521 15363801 10.1016/j.ceb.2004.07.007

[16] Irie K, Shimizu K, Sakisaka T, Ikeda W, Takai Y (2004) Roles and modes of action of nectins in cell-cell adhesion. Semin Cell Dev Biol 15:643–56 15561584 10.1016/j.semcdb.2004.09.002

[17] Inagaki M, Irie K, Ishizaki H, Tanaka-Okamoto M, Morimoto K, Inoue E, et al (2005) Roles of cell-adhesion molecules nectin 1 and nectin 3 in ciliary body development. Development 132:1525–1537 15728677 10.1242/dev.01697

[18] Barron MJ, Brookes SJ, Draper CE, Garrod D, Kirkham J, Shore RC, Dixon MJ (2008) The cell adhesion molecule nectin-1 is critical for normal enamel formation in mice. Hum Mol Genet 17:3509–20 10.1093/hmg/ddn243 18703497 PMC2572697

[19] Rikitake Y, Mandai K, Takai Y (2012) The role of nectins in different types of cell-cell adhesion. J Cell Sci 125:3713–3722 10.1242/jcs.099572 23027581

[20] Yoshida T, Miyoshi J, Takai Y, Thesleff I (2010) Cooperation of nectin-1 and nectin-3 is required for normal ameloblast function and crown shape development in mouse teeth. Dev Dyn 239:2558–69 10.1002/dvdy.22395 21038445

[21] Di Giovine P, Settembre EC, Bhargava AK, Luftig MA, Lou H, Cohen GH, et al (2011) Structure of herpes simplex virus glycoprotein D bound to the human receptor nectin-1. PLoS Pathog 7:e1002277 10.1371/journal.ppat.1002277 21980294 PMC3182920

[22] Stiles KM, Krummenacher C (2010) Glycoprotein D actively induces rapid internalization of two nectin-1 isoforms during herpes simplex virus entry. Virology 399:109–119 10.1016/j.virol.2009.12.034 20089288 PMC2830393

[23] Hall J V, Sun J, Slade J, Kintner J, Bambino M, Whittimore J, Schoborg R V (2014) Host nectin-1 is required for efficient Chlamydia trachomatis serovar E development. Front Cell Infect Microbiol 4:158 10.3389/fcimb.2014.00158 25414835 PMC4222120

[24] Deka S, Vanover J, Dessus-Babus S, Whittimore J, Howett MK, Wyrick PB, Schoborg R V. (2006) Chlamydia trachomatis enters a viable but non-cultivable (persistent) state within herpes simplex virus type 2 (HSV-2) co-infected host cells. Cell Microbiol 8:149–162 16367874 10.1111/j.1462-5822.2005.00608.x

[25] Phillips Campbell R, Kintner J, Whittimore J, Schoborg R V. (2012) Chlamydia muridarum enters a viable but non-infectious state in amoxicillin-treated BALB/c mice. Microbes Infect 14:1177–1185 10.1016/j.micinf.2012.07.017 22943883 PMC3654801

[26] Perry LL, Hughes S (1999) Chlamydial colonization of multiple mucosae following infection by any mucosal route. Infect Immun 67:3686–9 10377161 10.1128/iai.67.7.3686-3689.1999 PMC116566

[27] Ogita H, Rikitake Y, Miyoshi J, Takai Y (2010) Cell adhesion molecules nectins and associating proteins: Implications for physiology and pathology. Proc Jpn Acad Ser B Phys Biol Sci 86:621–9 20551598 10.2183/pjab.86.621 PMC3081173

[28] Sakamoto Y, Ogita H, Hirota T, Kawakatsu T, Fukuyama T, Yasumi M, et al (2006) Interaction of integrin alpha(v)beta3 with nectin. Implication in cross-talk between cell-matrix and cell-cell junctions. J Biol Chem 281:19631–44 16679515 10.1074/jbc.M600301200

[29] Wyrick PS, Davis CH, Wayner EA (1994) Chlamydia trachomatis does not bind to αβ1 integrins to colonize a human endometrial epithelial cell line cultured in vitro. Microb Pathog 17:159–166 7535373 10.1006/mpat.1994.1062

[30] Kumar Y, Valdivia RH (2008) Actin and intermediate filaments stabilize the Chlamydia trachomatis vacuole by forming dynamic structural scaffolds. Cell Host Microbe 4:159–69 10.1016/j.chom.2008.05.018 18692775 PMC2605408

[31] Yang C, Starr T, Song L, Carlson JH, Sturdevant GL, Beare PA, et al (2015) Chlamydial Lytic Exit from Host Cells Is Plasmid Regulated. MBio 6:e01648–15 10.1128/mBio.01648-15 26556273 PMC4659467

[32] Hybiske K, Stephens RS (2007) Mechanisms of host cell exit by the intracellular bacterium Chlamydia. Proc Natl Acad Sci U S A 104:11430–5 17592133 10.1073/pnas.0703218104 PMC2040915

[33] Kong FYS, Tabrizi SN, Fairley CK, Vodstrcil LA, Huston WM, Chen M, et al (2015) The efficacy of azithromycin and doxycycline for the treatment of rectal chlamydia infection: a systematic review and meta-analysis. J Antimicrob Chemother 70:1290–7 10.1093/jac/dku574 25637520

[34] Galen B, Cheshenko N, Tuyama A, Ramratnam B, Herold BC (2006) Access to nectin favors herpes simplex virus infection at the apical surface of polarized human epithelial cells. J Virol 80:12209–18 17005657 10.1128/JVI.01503-06 PMC1676285

[35] Noyce RS, Bondre DG, Ha MN, Lin L-T, Sisson G, Tsao M-S, Richardson CD (2011) Tumor cell marker PVRL4 (nectin 4) is an epithelial cell receptor for measles virus. PLoS Pathog 7:e1002240 10.1371/journal.ppat.1002240 21901103 PMC3161989

[36] LaFrance ME, Farrow MA, Chandrasekaran R, Sheng J, Rubin DH, Lacy DB (2015) Identification of an epithelial cell receptor responsible for Clostridium difficile TcdB-induced cytotoxicity. Proc Natl Acad Sci U S A 112:7073–8 10.1073/pnas.1500791112 26038560 PMC4460460

[37] Tanaka-Okamoto M, Hori K, Ishizaki H, Itoh Y, Onishi S, Yonemura S, et al (2011) Involvement of afadin in barrier function and homeostasis of mouse intestinal epithelia. J Cell Sci 124:2231–40 10.1242/jcs.081000 21652626 PMC3115770

[38] Darville T, Andrews CW, Laffoon KK, Shymasani W, Kishen LR, Rank RG (1997) Mouse strain-dependent variation in the course and outcome of chlamydial genital tract infection is associated with differences in host response. Infect Immun 65:3065–73 9234755 10.1128/iai.65.8.3065-3073.1997 PMC175432

[39] Linehan MM, Richman S, Krummenacher C, Eisenberg RJ, Cohen GH, Iwasaki A (2004) In Vivo Role of Nectin-1 in Entry of Herpes Simplex Virus Type 1 (HSV-1) and HSV-2 through the Vaginal Mucosa. J Virol 78:2530–2536 14963155 10.1128/JVI.78.5.2530-2536.2004 PMC369262

[40] Taylor JM, Lin E, Susmarski N, Yoon M, Zago A, Ware CF, et al (2007) Alternative entry receptors for herpes simplex virus and their roles in disease. Cell Host Microbe 2:19–28 18005714 10.1016/j.chom.2007.06.005 PMC2083283

[41] Hall JV, Schell M, Dessus-Babus S, Moore CG, Whittimore JD, Sal M, et al (2011) The multifaceted role of oestrogen in enhancing Chlamydia trachomatis infection in polarized human endometrial epithelial cells. Cell Microbiol 13:1183–99 10.1111/j.1462-5822.2011.01608.x 21615662

[42] Kintner J, Schoborg R V, Wyrick PB, Hall J V (2015) Progesterone antagonizes the positive influence of estrogen on Chlamydia trachomatis serovar E in an Ishikawa/SHT-290 co-culture model. Pathog Dis. 10.1093/femspd/ftv015 PMC4542640 25724891

[43] Singh S, Sheppard MC, Langman MJ (1993) Sex differences in the incidence of colorectal cancer: an exploration of oestrogen and progesterone receptors. Gut 34:611–5 8504960 10.1136/gut.34.5.611 PMC1374176

[44] Li CP, Ling C, Biancani P, Behar J (2012) Effect of progesterone on colonic motility and fecal output in mice with diarrhea. Neurogastroenterol Motil 24:392–e174 10.1111/j.1365-2982.2011.01875.x 22284724

